# Disease detected through screening is associated with superior survival outcomes in stage III colorectal cancer: a retrospective study in a Chinese high-volume cancer center

**DOI:** 10.1186/s12876-025-04294-z

**Published:** 2025-09-29

**Authors:** Fan Chen, Dakui Luo, Xun Jiang, Jiayu Chen, Ruoxin Zhang, Qingguo Li, Xinxiang Li

**Affiliations:** 1 Department of Colorectal Surgery, Fudan University Shanghai Cancer Center, 270 Dongan Road, Xuhui District, Shanghai, 200032 China; 2 Department of Oncology, Shanghai Medical College, Fudan University, Shanghai, China; 3 Jinling Hospital, Affiliated Hospital of Medical School, Nanjing University, Nanjing, China

**Keywords:** Locally advanced colorectal cancer, Screening, Survival outcome, Propensity score matching, Contributed equally

## Abstract

**Purpose:**

Colorectal cancer (CRC) screening reduces CRC mortality through early detection of cancer while decreasing incidence via removal of precancerous lesions (adenomas). Moreover, screening is associated with a reduction in mortality through detection of CRC in early stage. Yet it is unclear whether screening decrease mortality in locally advanced CRC. This study aims to investigate the pathological characteristics and survival outcomes of stage III CRC patients detected through screening. Notably in China and most developing countries where opportunistic screening predominates, understanding the stage-specific survival benefits could inform national screening policy optimization.

**Methods:**

This study involves a retrospective review of a cohort comprising 18,800 patients in Fudan University Shanghai Cancer Center, and a total of 4104 met the inclusion criteria. Propensity score matching (PSM) was conducted at a 1:4 ratio.

**Results:**

Compared with cancers not detected through screening (*n* = 3967, 96.7%), cancers detected through screening (*n* = 137, 3.3%) were more likely to be older, to have colon cancer, vascular invasion, perineural invasion, smaller tumors or to receive radical surgery without neoadjuvant therapy. After PSM, cancer detection by screening was associated with better overall survival (OS) and a non-significant trend toward improved disease-free survival (DFS) in stage III CRC (5-year OS: 83.4% vs. 69.7%, *P* = 0.005; 3-year DFS: 75.6% vs. 70.3%, *P* = 0.064). Multivariate Cox analysis demonstrated that cancer detection by screening was an independently protective factor for OS (HR: 0.517, 95% CI: 0.341–0.782, *P* = 0.002) and DFS (HR: 0.683, 95% CI: 0.475–0.982, *P* = 0.040) in stage III patients.

**Conclusion:**

The study demonstrates that screening-detected cancers reduce mortality and recurrence risks among stage III CRC patients. These findings emphasize that screening improves survival outcomes not only through early-stage detection, and highlight the urgent need to increase screening coverage. The results support incorporating survival metrics into cost-effectiveness evaluations of screening programs, providing policymakers with more nuanced evidence.

## Introduction

Colorectal cancer (CRC) is the third most common malignancy worldwide and represents the second leading cause of cancer-related mortality, resulting in a significant health burden [1]. Many countries have implemented a variety of CRC screening programs to address the increasing burden of this condition. CRC screening reduces CRC mortality through early detection of cancer while decreasing incidence via removal of precancerous lesions (adenomas) [2–4]. Moreover, screening is associated with a reduction in mortality through detection of CRC in early stage [5–8]. Depending on the different screening tests, screening can reduce mortality by 18–57% [9].

Most guidelines advocate for CRC screening in individuals aged 50 to 75 at general risk [10]. Nevertheless, there remains an insufficiency in evidence assessing the effectiveness of different screening tests [11].

Numerous studies have revealed that cancer detection by screening is associated with more favorable prognosis in CRC [4, 12–15]. These studies proposed that the survival benefits can be attributed primarily to the fact that cancers detected through screening tend to be identified at an earlier stage. However, it remains unclear whether screening decreases mortality in locally advanced CRC. Despite the statistical adjustments for staging through regression analysis in these studies, the potential presence of additional pathological factors beyond staging has generally been ignored.

The objective of this study was to analyze the pathological characteristics of stage III CRC detected through screening and to assess the influence of screening on the survival outcomes of locally advanced CRC by employing propensity score matching (PSM).

## Materials and methods

### Study population

A retrospective review was conducted using data from 18,800 patients who underwent surgical intervention for CRC at Fudan University Shanghai Cancer Center (FUSCC) in Shanghai, China, from January 1, 2008, to December 31, 2019. Patient information was collected prospectively to establish a comprehensive database at FUSCC.

**Fig. 1:**
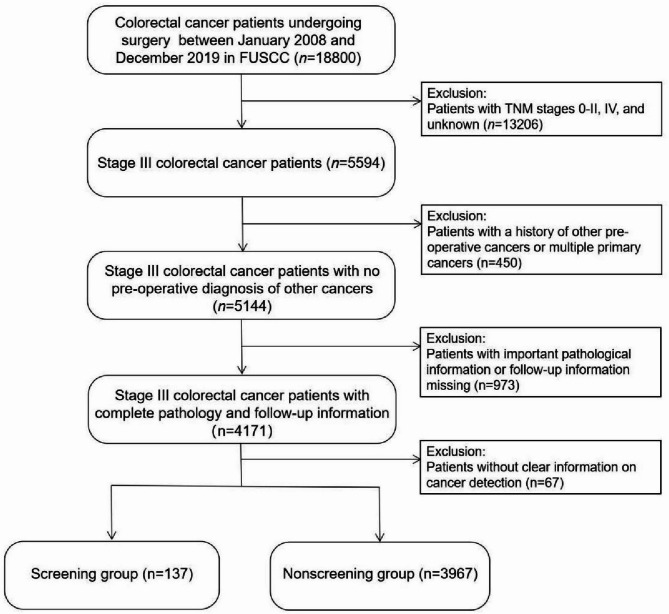
Flow chart of patient selection process

The inclusion criteria were as follows: patients who underwent radical surgery for CRC, had confirmed stage III CRC through postoperative pathology, had no prior history of cancer before surgery, and had clear cancer detection information. Patients who were detected through any screening test were categorized into the screening group, while patients who were detected based on symptoms formed the non-screening group. Figure 1 demonstrates the detailed process of patient inclusion and exclusion.

Data extracted from the database included the following patient information: age at diagnosis, gender, tumor location, neoadjuvant therapy, surgical procedures, histologic type, differentiation, vascular invasion, perineural invasion, pathological T stage, pathological N stage, and survival outcomes. The study protocol was approved by The Ethical Committee and Institutional Review Board of FUSCC. For variables with ‘unknown’ categories (e.g., differentiation, T stage), these were retained as a separate group in all analyses. This classification represents cases where the pathological feature could not be definitively assessed and is a clinically recognized, non-random occurrence.

In this study, screening-detected cases were identified through opportunistic screening, which represents the predominant screening approach in the Chinese context. The screening modalities primarily included colonoscopy and fecal immunochemical test (FIT). Participants undergoing screening were typically asymptomatic individuals presenting for routine health check-ups or those identified as high-risk based on clinical assessment (e.g., family history of CRC, personal history of polyps, or concerning symptoms warranting diagnostic evaluation). This approach reflects the current real-world screening practices in China, where organized population-based screening programs are not yet universally implemented.

### Postoperative follow-up

Survival data were collected through a combination of follow-up activities, including reviewing medical records, conducting telephonic follow-up, and retrieving data from death registries. In this study, the primary survival outcomes assessed were overall survival (OS) and disease-free survival (DFS). OS was defined as the duration from the initial treatment to either death from any cause or the last recorded follow-up. DFS was defined as the duration from the initial treatment to the occurrence of local or distant relapse, death from any cause, or the date of the last recorded follow-up.

### Statistical analysis

All statistical analyses were conducted using SPSS version 27.0 and R version 4.3.1. *χ*^2^ test was used to compare nominal variables between two distinct groups, while the Mann-Whitney *U* test was utilized to compare ordered multiclass variables. Kaplan-Meier analysis was used to assess and compare OS and DFS between the two groups. To mitigate bias in the sample selection process, PSM was performed at a 1:4 ratio using the MatchIt package in R with a caliper value of 0.02 to compare the OS and DFS of the two groups. The standardized mean differences (SMDs) for all baseline characteristics before and after matching were calculated using the tableone package. A balanced distribution between the groups after PSM was considered acceptable if the absolute SMD for each variable was less than 0.1. Baseline characteristics, including age at diagnosis, gender, tumor location, neoadjuvant therapy, surgical procedures, histologic type, differentiation, vascular invasion, perineural invasion, pathological T stage, and pathological N stage, were matched between the groups. Multivariate Cox regression analysis was used to identify independent prognostic factors. A *P* < 0.05 was considered as statistically significant.

## Results

The study included a total of 4,104 cases. Among these, 137 (3.3%) cases were detected by screening, while 3,967 (96.7%) cases were not detected by screening, forming the screening and non-screening groups.

The baseline characteristics of both groups were as follows (Table 1). Compared to patients in the non-screening group, patients in the screening group had a significantly lower proportion of rectal cancers (35.8% vs. 56.1%, *P* < 0.001) and a correspondingly higher proportion of colon cancers (left-sided: 32.1% vs. 21.8%; right-sided: 29.9% vs. 20.9%). The screening group also showed a significantly higher proportion of patients aged ≥ 50 years (86.9% vs. 76.1%, *P* = 0.004), lower rates of neoadjuvant therapy (2.9% vs. 10.5%, *P* = 0.004), and higher prevalence of both vascular invasion (53.3% vs. 41.9%, *P* = 0.008) and perineural invasion (42.3% vs. 33.5%, *P* = 0.031). Additionally, the screening group had a different T stage distribution with higher proportions of T3 (51.8% vs. 32.8%) and lower proportions of T4 (32.8% vs. 52.7%) diseases (*P* < 0.001). Two groups were well-balanced in terms of other characteristics, including gender, surgical approach, histologic type, differentiation degree, and N stage (all *P* > 0.05).

**Table 1:** Pathological characteristics of patients before and after propensity score matching in this study

Variable	All cohort before PSM			Matched cohort after PSM		
	Screening (*n* = 137)	Nonscreening (*n* = 3967)	*P* value	Screening (*n* = 137)	Nonscreening (*n* = 548)	*P* value
Gender			0.688			0.673
Male	77 (56.2%)	2298 (57.9%)		77 (56.2%)	297 (54.2%)	
Female	60 (43.8%)	1669 (42.1%)		60 (43.8%)	251 (45.8%)	
Age (years)			0.004			0.551
< 50	18 (13.1%)	948 (23.9%)		18 (13.1%)	61 (11.1%)	
≥ 50	119 (86.9%)	3019 (76.1%)		119 (86.9%)	487 (88.9%)	
Location			< 0.001			0.328
Rectum	49 (35.8%)	2226 (56.1%)		49 (35.8%)	224 (40.9%)	
Left-sided colon	44 (32.1%)	866 (21.8%)		44 (32.1%)	142 (25.9%)	
Right-sided colon	41 (29.9%)	830 (20.9%)		41 (29.9%)	162 (29.6%)	
Unknown	3 (2.2%)	45 (1.4%)		3 (2.2%)	20 (3.6%)	
Neoadjuvant therapy			0.004			0.907
No	133 (97.1%)	3550(89.5%)		133 (97.1%)	533 (97.3%)	
Yes	4 (2.9%)	417 (10.5%)		4 (2.9%)	15 (2.7%)	
Surgical procedures			0.190			0.529
Open	108 (78.8%)	3297 (83.1%)		108 (78.8%)	445 (81.2%)	
Laparoscopic	29 (21.2%)	670 (16.9%)		29 (21.2%)	103 (18.8%)	
Histologic type			0.632			0.676
Adenocarcinoma	119 (86.9%)	3379 (85.2%)		119 (86.9%)	482 (88.0%)	
Mucinous	12 (8.8%)	453 (11.4%)		12 (8.8%)	53 (9.7%)	
Signet ring cell	6 (4.4%)	135 (3.4%)		6 (4.4%)	13 (2.4%)	
Differentiation			0.607			0.609
Poor	40 (29.2%)	1175 (29.6%)		40 (29.2%)	144 (26.3%)	
Moderate	89 (65.0%)	2553 (64.4%)		89 (65.0%)	380 (69.3%)	
well	3 (2.2%)	24 (0.6%)		3 (2.2%)	6 (1.1%)	
Unknown	5 (3.6%)	215 (5.4%)		5 (3.6%)	18 (3.3%)	
Vascular invasion			0.008			0.422
Negative	64 (46.7%)	2306 (58.1%)		64 (46.7%)	277 (50.5%)	
Positive	73 (53.3%)	1661 (41.9%)		73 (53.3%)	271 (49.5%)	
Perineural invasion			0.031			0.786
Negative	79 (57.7%)	2639 (66.5%)		79 (57.7%)	323 (58.9%)	
Positive	58 (42.3%)	1328 (33.5%)		58 (42.3%)	225 (41.1%)	
T stage			< 0.001			0.963
0–2	21 (15.3%)	483 (12.2%)		21 (15.3%)	96 (17.5%)	
3	71 (51.8%)	1301 (32.8%)		71 (51.8%)	264 (48.2%)	
4	45 (32.8%)	2090 (52.7%)		45 (32.8%)	188 (34.3%)	
Unknown	0 (0.0%)	93 (2.3%)		0 (0.0%)	0 (0.0%)	
N stage			0.533			0.418
1	87 (63.5%)	2621 (66.1%)		87 (63.5%)	368 (67.2%)	
2	50 (36.5%)	1346 (33.9%)		50 (36.5%)	180 (32.8%)	

**Fig. 2:**
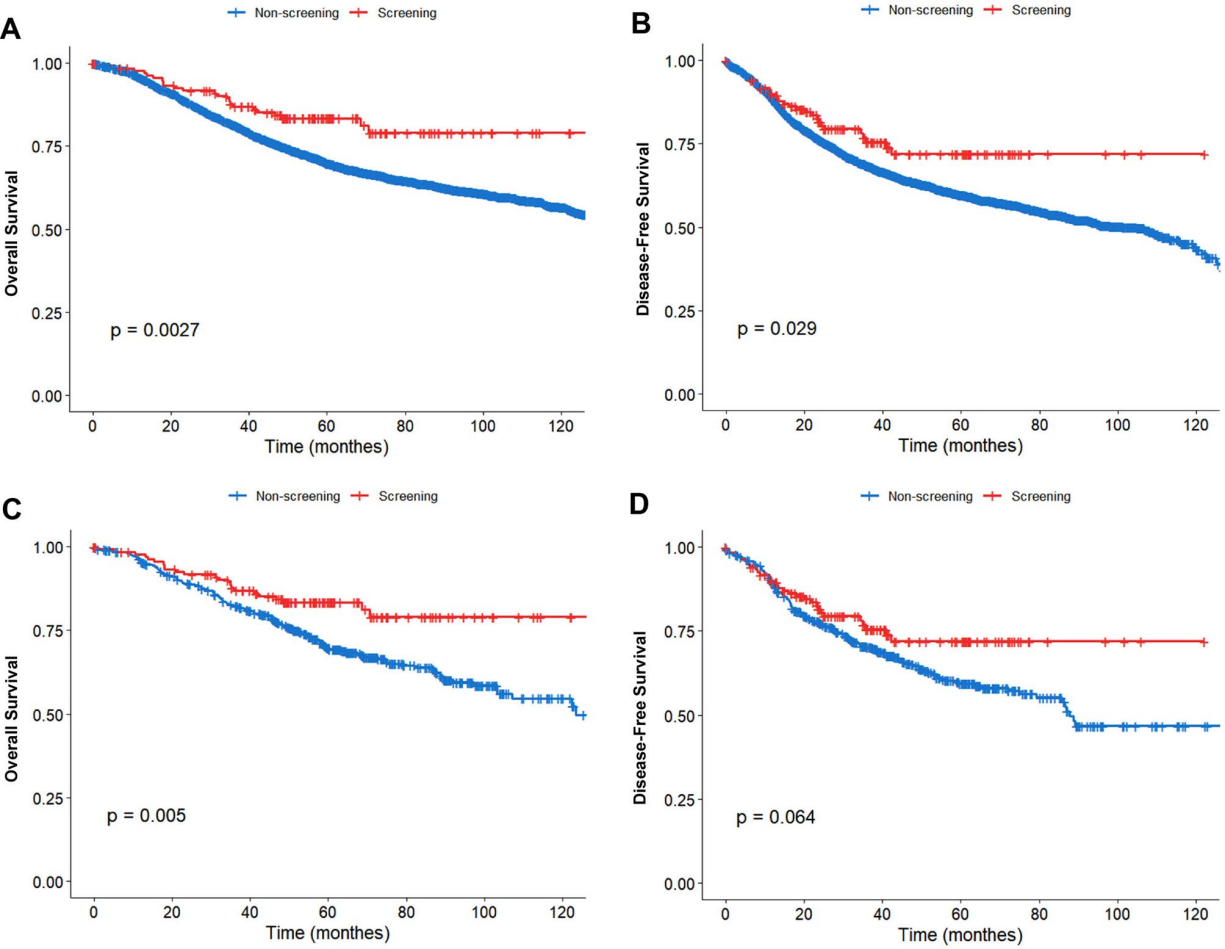
Prognostic analysis of patients before and after propensity score matching. Kaplan-Meier analysis of overall survival (OS) and disease-free survival (DFS) stratified by mode of cancer detection (screening-detected vs. non-screened). **A** OS before matching; **B** DFS before matching; **C** OS after 1:4 propensity score matching; **D** DFS after matching

The median follow-up duration for the entire cohort was 58.2 months. Throughout this period, 1497 (36.5%) patients experienced recurrence and 1317 (32.1%) patients died. Patients in the screening group exhibited significantly better OS (5-year OS: 83.4% vs. 69.9%, *P* = 0.003; Fig. 2A) and DFS (3-year DFS: 75.6% vs. 68.5%, *P* = 0.029; Fig. 2B) compared to those in the non-screening group.

**Fig. 3:**
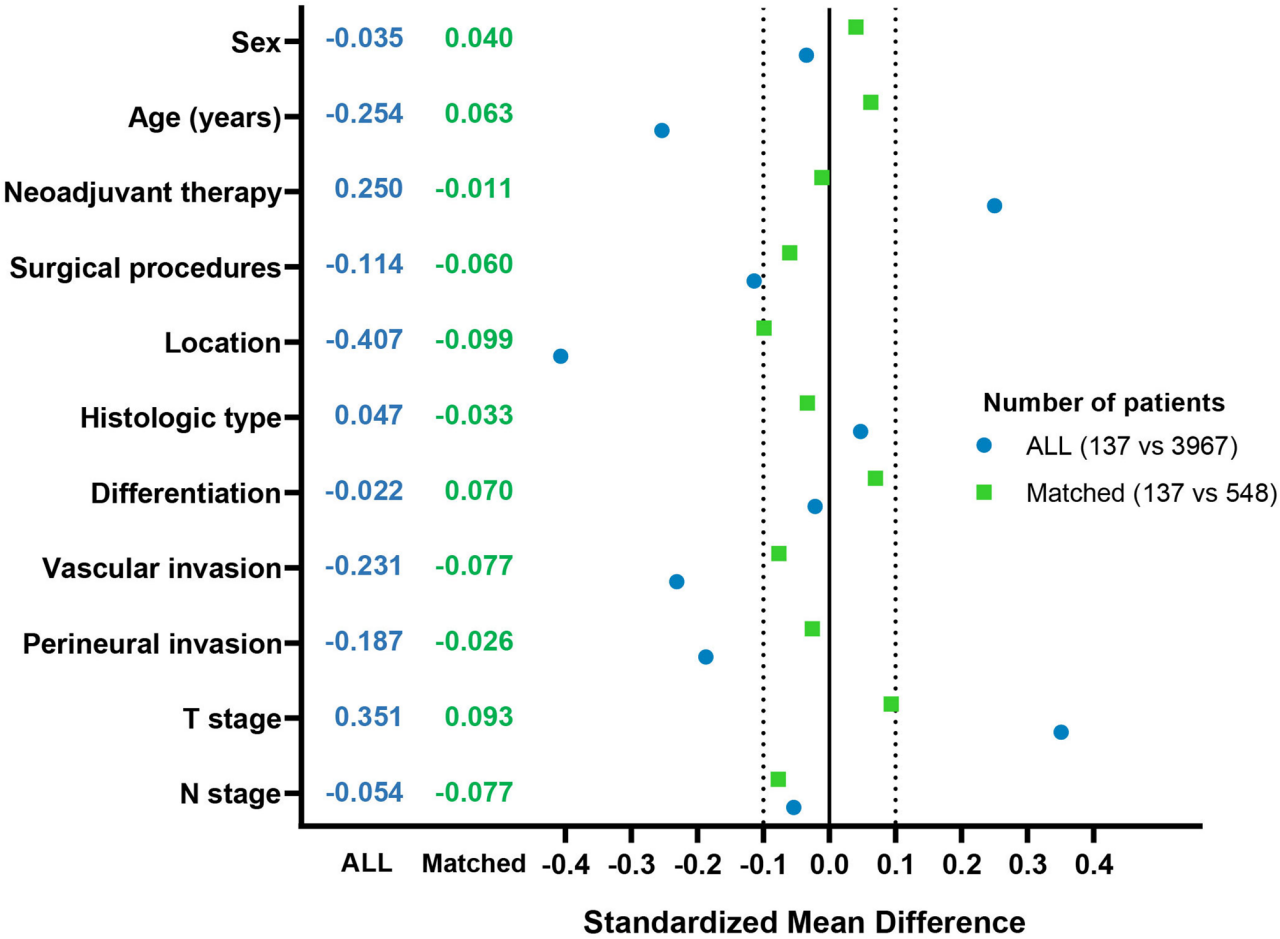
Assessment of covariate balance before and after propensity score matching

To minimize potential confounding, we performed a 1:4 propensity score matching, resulting in a well-balanced cohort of 685 patients (screening group: *n* = 137; non-screening group: *n* = 548) for further analysis. The balance of all covariates after matching is shown in Table 1 and visualized via standardized mean differences in Fig. 3. In this matched cohort, the survival advantage for the screening group persisted. OS remained significantly better (5-year OS: 83.4% vs. 69.7%, *P* = 0.005; Fig. 2C), representing a 13.7% absolute improvement. A consistent, though not statistically significant, trend towards improved DFS was also observed (3-year DFS: 75.6% vs. 70.3%, Log-rank *P* = 0.064; Fig. 2D), with a 5.3% absolute improvement.

In multivariate Cox analysis, after adjusting for potential confounders, screening-detected cancer was independently associated with significantly reduced risks of both mortality (HR: 0.517, 95% CI: 0.341–0.782, *P* = 0.002 for OS) and recurrence (HR: 0.683, 95% CI: 0.475–0.982, *P* = 0.040 for DFS). Besides the mode of detection, older age (OS HR: 1.029, 95% CI: 1.024–1.034, *P* < 0.001; DFS HR: 1.012, 95% CI: 1.007–1.016, *P* < 0.001), left-sided colon location (OS HR: 0.857, 95% CI: 0.739–0.995, *P* = 0.043), right-sided colon location (DFS HR: 1.211, 95% CI: 1.064–1.378, *P* = 0.004), neoadjuvant therapy (OS HR: 2.051, 95% CI: 1.707–2.464, *P* < 0.001; DFS HR: 1.876, 95% CI: 1.570–2.241, *P* < 0.001), signet ring cell histology (OS HR: 1.755, 95% CI: 1.356–2.271, *P* < 0.001), moderate differentiation (OS HR: 0.674, 95% CI: 0.593–0.766, *P* < 0.001; DFS HR: 0.787, 95% CI: 0.802–0.882, *P* < 0.001), vascular invasion (OS HR: 1.320, 95% CI: 1.171–1.487, *P* < 0.001; DFS HR: 1.294, 95% CI: 1.156–1.448, *P* < 0.001), perineural invasion (OS HR: 1.548, 95% CI: 1.377–1.740, *P* < 0.001; DFS HR: 1.511, 95% CI: 1.353–1.687, *P* < 0.001), advanced T stage (T3/T4), and N2 stage (OS HR: 1.524, 95% CI: 1.354–1.715, *P* < 0.001; DFS HR: 1.460, 95% CI: 1.306–1.632, *P* < 0.001) were also identified as independent prognostic factors for survival outcomes (Tables 2 and 3).

**Table 2:** Univariate and multivariate Cox analyses of risk factors for overall survival

Variable	Univariate analysis HR (95% CI)	*P* value	Multivariate analysis HR (95% CI)	*P* value
Mode of detection				
Non-screening	Reference		Reference	
Screening	0.537 (0.355–0.811)	0.003	0.517 (0.341–0.782)	0.002
Gender				
Female	Reference		NI	
Male	0.933 (0.836–1.041)	0.216		
Age (years)	1.020 (1.015–1.025)	0.003	1.029 (1.024–1.034)	< 0.001
Location				
Rectum	Reference		Reference	
Left-sided colon	0.811 (0.702–0.936)	0.004	0.857 (0.739–0.995)	0.043
Right-sided colon	1.147 (1.005–1.308)	0.042	1.136 (0.989–1.305)	0.072
Unknown	/		/	
Neoadjuvant therapy				
No	Reference		Reference	
Yes	1.487 (1.266–1.746)	< 0.001	2.051 (1.707–2.464)	< 0.001
Surgical procedures				
Open	Reference		NI	
Laparoscopic	0.966 (0.829–1.126)	0.660		
Histologic type				
Adenocarcinoma	Reference		Reference	
Mucinous	1.200 (1.021–1.410)	0.027	0.999 (0.838–1.192)	0.995
Signet ring cell	2.507 (1.977–3.179)	< 0.001	1.755 (1.356–2.271)	< 0.001
Differentiation				
Poor	Reference		Reference	
Moderate	0.550 (0.491–0.617)	< 0.001	0.674 (0.593–0.766)	< 0.001
Well	0.617 (0.319–1.194)	0.152	0.984 (0.506–1.915)	0.963
Unknown	0.879 (0.697–1.109)	0.879	1.113 (0.846–1.464)	0.444
Vascular invasion				
Negative	Reference		Reference	
Positive	1.654 (1.484–1.843)	< 0.001	1.320 (1.171–1.487)	< 0.001
Perineural invasion				
Negative	Reference		Reference	
Positive	1.735 (1.555–1.937)	< 0.001	1.548 (1.377–1.740)	< 0.001
T stage				
0–2	Reference		Reference	
3	1.826 (1.461–2.283)	< 0.001	1.357 (1.079–1.707)	0.009
4	2.083 (1.689–2.569)	< 0.001	1.754 (1.414–2.176)	< 0.001
Unknown	/		/	
N stage				
1	Reference		Reference	
2	1.772 (1.589–1.977)	< 0.001	1.524 (1.354–1.715)	< 0.001

*NI* Not Included in multivariate analysis

**Table 3:** Univariate and multivariate Cox analyses of risk factors for disease-free survival

Variable	Univariate analysis HR (95% CI)	*P* value	Multivariate analysis HR (95% CI)	*P* value
Mode of detection				
Non-screening	Reference		Reference	
Screening	0.670 (0.467–0.962)	0.030	0.683 (0.475–0.982)	0.040
Gender				
Female	Reference		NI	
Male	1.089 (0.982–1.208)	0.105		
Age (years)	1.006 (1.002–1.011)	0.003	1.012 (1.007–1.016)	< 0.001
Location				
Rectum	Reference		Reference	
Left-sided colon	0.877 (0.767–1.004)	0.056	0.933 (0.812–1.072)	0.328
Right-sided colon	1.188 (1.050–1.344)	0.006	1.211 (1.064–1.378)	0.004
Unknown	/		/	
Neoadjuvant therapy				
No	Reference		Reference	
Yes	1.483 (1.270–1.733)	< 0.001	1.876 (1.570–2.241)	< 0.001
Surgical procedures				
Open	Reference		NI	
Laparoscopic	0.925 (0.797–1.075)	0.309		
Histologic type				
Adenocarcinoma	Reference		NI	
Mucinous	1.051 (0.899–1.228)	0.531		
Signet ring cell	1.812 (1.428–2.299)	< 0.001		
Differentiation				
Poor	Reference		Reference	
Moderate	0.643 (0.577–0.716)	< 0.001	0.787 (0.702–0.882)	< 0.001
Well	0.488 (0.243–0.980)	0.044	0.782 (0.387–1.580)	0.494
Unknown	0.947 (0.760–1.180)	0.628	1.123 (0.862–1.462)	0.389
Vascular invasion				
Negative	Reference		Reference	
Positive	1.563 (1.412–1.729)	< 0.001	1.294 (1.156–1.448)	< 0.001
Perineural invasion				
Negative	Reference		Reference	
Positive	1.707 (1.540–1.893)	< 0.001	1.511 (1.353–1.687)	< 0.001
T stage				
0–2	Reference		Reference	
3	1.821 (1.480–2.241)	< 0.001	1.408 (1.136–1.744)	0.002
4	1.948 (1.606–2.363)	< 0.001	1.629 (1.335–1.988)	< 0.001
Unknown	/		/	
N stage				
1	Reference		Reference	
2	1.689 (1.524–1.871)	< 0.001	1.460 (1.306–1.632)	< 0.001

## Discussion

Over the past two decades, there has been a significant decline in the incidence and mortality of CRC in many countries due to the widespread implementation of CRC screening. Within Europe, countries with long-standing screening programs and comprehensive population coverage have experienced substantial decreases in the incidence of CRC [16]. Similarly, in the United States, the incidence of CRC decreased by nearly 40% as the proportion of individuals aged 50 and above who underwent recommended screening tests increased from 38% in 2000 to 66% in 2018 [17]. However, in more developing countries such as China, CRC screening coverage is extremely low. The incidence and mortality of CRC continue to increase in China [18]. Low coverage of CRC screening persists due to the scarcity and uneven allocation of medical resources per capita. A major cohort study involving 3,165,088 participants across 18 provinces in China revealed that only 2.81% of individuals had undergone colonoscopy screening between 2013 and 2021. Our study revealed that only 3.3% of stage III CRC cases were detected through screening, while the majority of locally advanced cases were not detected through screening.

Our study identified specific characteristics among the CRC patients detected through screening, such as older age, colon cancer, adenocarcinoma, vascular invasion, perineural invasion, smaller tumors and to receive radical surgery without neoadjuvant therapy. Previous studies have shown that cancers detected through screening tend to be diagnosed at earlier stages, which is also a crucial factor contributing to the survival benefits observed in screened patients [5, 15, 19, 20]. In our cohort, the association between screening and staging was primarily observed in T staging, rather than N staging. This is partly due to the early detection of disease through screening, as well as the fact that larger tumors often present with more noticeable symptoms.

A paradoxical finding of our study was that screening-detected cancers exhibited higher rates of vascular and perineural invasion compared to non-screened cancers. We propose that the compositional difference in tumor location between the two groups explains this apparent contradiction. Screening is more likely to detect asymptomatic proximal colon cancers. In contrast, rectal cancers often produce early symptoms (e.g., hematochezia, tenesmus), leading to their prompt clinical diagnosis and consequent overrepresentation in the nonscreening group [21]. Given that rectal cancers demonstrate a higher intrinsic propensity for vascular and perineural invasion compared to colon cancers [22, 23], the underrepresentation of these more aggressive rectal tumors in the screening-detected group provides a logical explanation for the observed pattern. The most critical evidence, however, comes from our multivariate analysis, which demonstrated that after rigorously adjusting for tumor location and the presence of vascular/perineural invasion (among other factors), screening detection remained an independent predictor of improved survival (OS HR: 0.517, 95% CI: 0.341–0.782, *P* = 0.002; DFS HR: 0.683, 95% CI: 0.475–0.982, *P* = 0.040).

The substantially enhanced survival of patients with screen-detected cancers cannot be solely attributed to earlier diagnosis. Even after adjusting for confounding factors using PSM and Cox analysis, a significant survival benefit persisted. While some researchers have hypothesized that the survival advantage in screening-detected patients might be explained if non-screened patients had more aggressive tumor characteristics [15], our study revealed no statistically significant discrepancies in histologic type and differentiation between cancers detected by screening and non-screening. Similarly, although screening might identify patients with heightened health consciousness and favorable socioeconomic characteristics [24–26], previous research adjusting for educational factors still found consistent screening benefits [27], suggesting that these factors alone cannot explain the observed survival advantage.

Beyond these considerations, we propose that several clinical mechanisms may contribute to the superior outcomes of screening-detected cancers. The lead time gained through screening allows for a more comprehensive diagnostic workup and optimized treatment planning rather than urgent intervention. This additional time enables better patient preparation, including nutritional optimization and management of comorbidities, which may enhance tolerance to aggressive treatments. Furthermore, the absence of emergency presentations may facilitate higher rates of complete (R0) resections and reduce postoperative complications. Patients identified through screening also enter a more structured healthcare pathway from diagnosis, potentially resulting in more consistent adherence to treatment protocols and surveillance schedules. The less advanced disease state at detection may also allow for better preservation of immune function and performance status, enabling patients to receive and complete optimal multimodal therapy.

It is crucial to consider the potential impact of lead time bias when evaluating screening benefits. However, given our extended follow-up period exceeding 60 months, the influence of lead time bias was likely minimized [28–30]. Our findings align with recent research demonstrating that screened CRC patients experience substantially lower recurrence rates [31], further reinforcing the critical significance of screening.

First, as a specialized cancer center, we tend to attract patients with more advanced cancers from various regions, which introduces selection bias when applying our findings to a wider general population. Second, our findings reflect the context of opportunistic rather than organized population-based screening, which typically involves populations that may differ in risk profiles, health-seeking behaviors, and socioeconomic status - factors further compounded by the geographic and economic disparities inherent in China’s healthcare landscape. Third, while PSM was employed to minimize confounding, the sample size of the screening-detected group remained limited, which may have reduced the statistical power of our analyses. Additionally, we cannot exclude residual confounding from unmeasured factors such as health literacy, treatment adherence, and socioeconomic status.

Despite these limitations, our findings carry significant implications for CRC screening policy, particularly in settings with limited per capita resource availability like China. The demonstrated survival benefit for even stage III screen-detected cancers strengthens the economic and public health argument for expanding screening efforts. Given the constraints of large-scale colonoscopy implementation, a stepped approach utilizing FIT as an initial, widespread primary screening tool followed by colonoscopy confirmation only for positive cases could maximize population coverage and efficient resource allocation. Furthermore, initially prioritizing high-risk populations represents a pragmatic and impactful policy to achieve significant public health benefits while building infrastructure for broader future expansion.

Future research should prioritize detailed analysis of treatment timelines, completeness of surgical resection, adherence to adjuvant therapy protocols, and comprehensive documentation of postoperative complications to elucidate the relative contributions of these clinical factors to the screening survival benefit. Larger-scale, multi-center studies incorporating geographic and socioeconomic data are warranted to confirm these findings across diverse healthcare settings.

## Conclusion

In conclusion, our study demonstrates that screening-detected cancers reduce mortality and recurrence risks among stage III CRC patients. These findings emphasize that the survival benefits of screening extend beyond early-stage detection, and highlight the urgent need to increase screening coverage. The results support incorporating survival metrics into cost-effectiveness evaluations of screening programs, providing policymakers with more nuanced evidence. Future multi-center studies in general populations are needed to validate these findings.

## Data Availability

The data that support the findings of this study are available from the corresponding author upon reasonable request.

## Abbreviations

CI: Confidence Interval
CRC: Colorectal Cancer
DFS: Disease-Free Survival
FIT: Fecal Immunochemical Test
FUSCC: Fudan University Shanghai Cancer Center
HR: Hazard Ratio
OS: Overall Survival
PSM: Propensity Score Matching
TNM: Tumor Node Metastasis
